# Importance of humidity for characterization and communication of dangerous heatwave conditions

**DOI:** 10.1038/s41612-023-00346-x

**Published:** 2023-04-27

**Authors:** Ivana Cvijanovic, Malcolm N. Mistry, James D. Begg, Antonio Gasparrini, Xavier Rodó

**Affiliations:** 1 Barcelona Institute for Global Health - ISGLOBAL, Doctor Aiguader 88, 08003 Barcelona, Spain; 2 Department of Public Health, Environments and Society, London School of Hygiene & Tropical Medicine, 15-17 Tavistock Place, WC1H 9SH London, United Kingdom; 3 Department of Economics, Ca’ Foscari University of Venice, Cannaregio 873/b, 30121 Venice, Italy; 4 The University of Manchester, Department of Earth and Environmental Sciences, Oxford Road, M13 9PL Manchester, United Kingdom; 5 The Centre on Climate Change & Planetary Health, London School of Hygiene & Tropical Medicine, 15-17 Tavistock Place, WC1H 9SH London, United Kingdom; 6 Centre for Statistical Methodology, London School of Hygiene & Tropical Medicine, 15-17 Tavistock Place, WC1H 9SH London, United Kingdom; 7 ICREA, Passeig Lluís Companys 23, 08010 Barcelona, Spain

## Abstract

Heatwaves are one of the leading causes of climate-induced mortality. Using the examples of recent heatwaves in Europe, the United States and Asia, we illustrate how the communication of dangerous conditions based on temperature maps alone can lead to insufficient societal perception of health risks. Comparison of maximum daily values of temperature with physiological heat stress indices accounting for impacts of both temperature and humidity, illustrates substantial differences in geographical extent and timing of their respective peak values during these recent events. This signals the need to revisit how meteorological heatwaves and their expected impacts are communicated. Close collaboration between climate and medical communities is needed to select the best heat stress indicators, establish them operationally, and introduce them to the public.

npj Climate and Atmospheric Science (2023) 6:33;

## Introduction

Heatwaves are typically defined as rare or unusually warm events for a given location and time of year, that exceed a specific temperature threshold for several consecutive days. The threshold that would classify a meteorological event as an extreme heatwave is therefore location-specific and varies around the globe. Due to climate change, thresholds for extreme temperature events are being redefined: for example, in 2022, the UK Met Office switched to using warmer 1991–2020 climatology for defining extreme temperature departures^1^. While the regional thresholds for classifying an event as an extreme heatwave are becoming higher, the human body's physical thresholds of tolerance to heat remain constant (i.e. there has been no evidence of a recent evolutionary adaptation that would allow humans to withstand or survive higher heat stress levels than before).

From ‘climate’ and ‘epidemiological’ perspectives, heatwaves are most commonly investigated using near-surface air temperature, also referred to as dry-bulb temperature. Traditionally, climate studies investigating future changes in extreme heatwaves used projections of maximum daily temperatures^2–7^, deriving various statistical indicators related to the magnitude, frequency or duration of the temperature-defined heatwaves. Likewise, meteorological maps used by the media to communicate forthcoming and ongoing heatwaves most commonly show the expected sub-daily or daily maximum temperatures^8,9^. Epidemiological studies investigating the links between the heatwaves and mortality use the near-surface air temperature as the key climate predictor for explaining heat-related outcomes^10^. From a physiological perspective, however, changes in temperature alone are not considered sufficient to explain the stress that a given extreme warm weather event can exert on the human body. This is because meteorological factors like the humidity of the surrounding air, the prevailing wind conditions and the length of previous thermal exposure can all have a large impact on how a given heatwave affects human health and the dangers it is likely to pose. For example, an air temperature of 37 °C can be perceived as uncomfortable for humans in low humidity, but in combination with high humidity and no wind, it creates conditions under which the human body cannot cool down, thereby potentially posing detrimental health risks.

With the aim of relating the impact of meteorological conditions on the human body and defining the limits dangerous for human health, the climate - health community has focused on the development of a number of heat-stress indices – humidex (Hu), apparent temperature (AT), wet bulb globe temperature (WBGT), heat index (HI) and universal thermal climate index (UTCI), amongst others - that incorporate the impacts of several meteorological variables (most notably humidity) in their assessment of associated health risks^11–13^. However, one of the major difficulties in characterizing dangerous heatwave conditions arises from the fact that it is not possible to conduct controlled experiments on a wider population that would investigate the onset of the most dangerous health outcomes under extreme heat conditions. The scientific community has thus often had to rely on using actual heatwave occurrences to try to extract such information^14^. Critical thresholds have also been suggested based on the laws of physics. For example, at 35 °C wet-bulb temperature (WBT), evaporation is not possible and cooling through sweating would not be effective, leading to the suggestion that this is the upper threshold for human survival in humid heat^15^. However, a recent study that carried out several controlled experiments on human response to heat concluded that a physics-only definition leads to a substantial overestimation of human health thresholds. Actual monitoring of heat stress levels in human subjects has shown that the human body can start to experience serious strain at 28 °C WBT, with 31 °C WBT representing the critical limit for adverse heat-related health effects in humid environments^16^. This marks a significant downward revision of human tolerance to humid heat. An important question that remains unanswered is how much these thresholds vary within populations and between different climate zones.

Encouragingly, global climate change studies have been including heat stress metrics in their analyses^7,14,17,18,19,20^ and this remains a rapidly developing field. One major milestone in this direction is the creation of new, publicly available datasets of the key heat stress indices over the historical period and in future climate projections^12,21^. A number of meteorological services around the world have implemented country-specific heat indices in the creation of heatwave alerts (e.g., humidex in Canada, UTCI in Germany, HI in the USA). However, the actual messaging of heatwave danger through news and media is still mostly linked to the maximum temperatures and rarely includes information on the expected heat indices values. Since the public is generally not well informed about the heat indices and their meaning, reporting temperatures seems like the best practice^22^. However, depending on the humidity, an event of 36 °C at a given location can in one instance be very uncomfortable and in another, dangerous. In these two cases, without setting two different alert levels and clearly communicating the substantially different health impacts expected, local populations' perception of the different levels of danger associated with the two 36 °C events may easily be lost. This is especially important when considering so-called ‘moist heatwaves’^23^ that are expected to increase in frequency with climate change. Moist heatwaves can make the conditions around the temperatures previously experienced as safe at a given location (during dry heatwaves) become dangerous.

Using examples of recent record-breaking heatwaves (the 2019 European heatwave, 2021 western United States heatwave, 2022 US mid-west heatwave and 2022 India-Pakistan heatwave), we illustrate the remarkable geographical differences between perceived heatwave danger when using maximum daily temperatures compared to the maximum daily values of two selected heatwave indices (humidex and indoor wet bulb globe temperature) that incorporate impacts of both temperature and humidity. These comparisons provide important insights as to how societal perception of dangerous heatwave conditions based on shortterm exposure to high temperatures alone can fail to grasp the geographical extent and timing of conditions dangerous for human health.

### June 2019 European heatwave

**Fig. 1:**
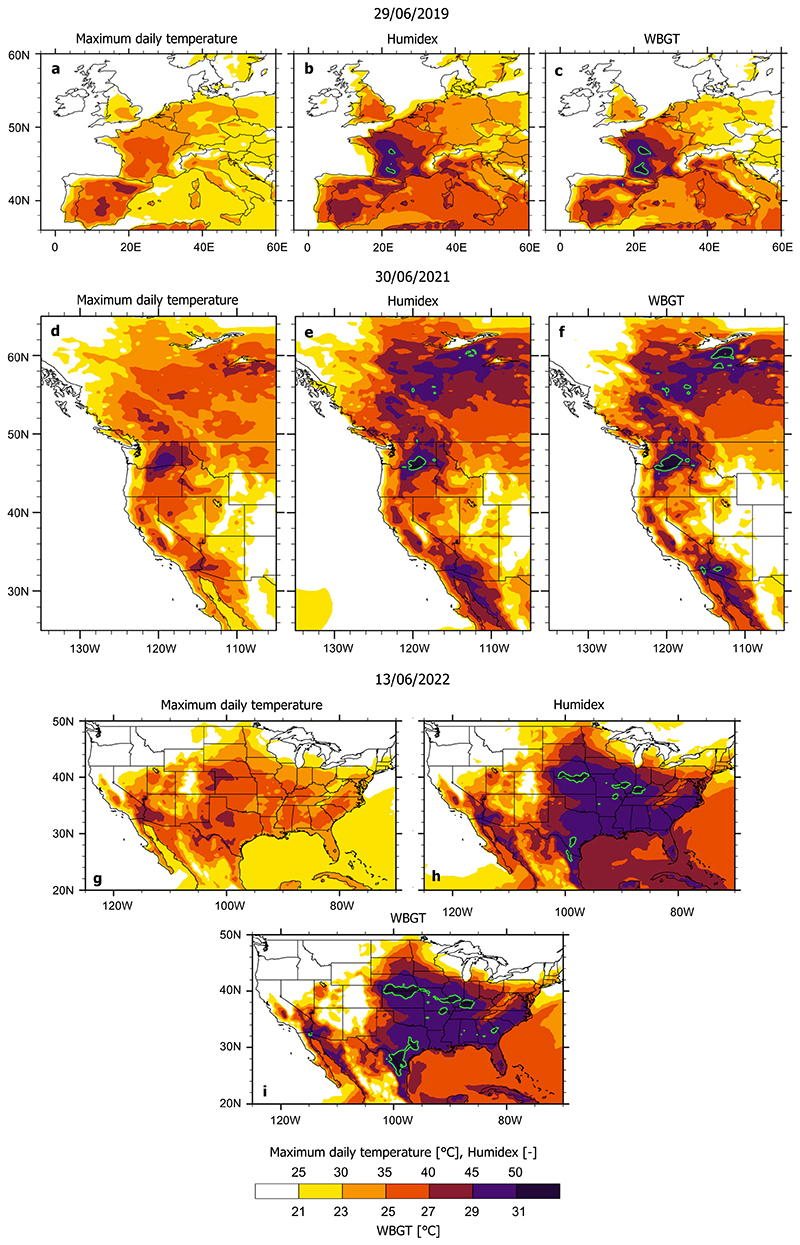
Maximum daily temperature [°C], humidex [unitless] and indoor Wet Bulb Globe Temperature (WBGT) [°C] during the selected days of three major heatwaves. **a-c** June 29th 2019 western Europe heatwave; **d-f** June 30th 2021 western United States heatwave; and **g-i** June 13th 2022 United States Midwest heatwave. Green contours indicate regions with humidex above 50 and indoor WBGT above 31 °C, where the critical threshold values defining dangerous heat stress conditions were exceeded (see Methods).

In late June of 2019, the first of the two major heatwaves of the year hit the European continent, breaking a national all-time high-temperature record for France^24^ on June 28. An interesting weather pattern emerged in the following days, as the temperatures in France appeared to decline marginally. On June 29, the maximum daily temperatures in Europe were observed in central and northeastern Spain (Fig. 1a). However, the heat indices maxima (shown by green contours and suggesting the conditions dangerous for human health) prevailed in central and southern France (Fig. 1b, c). A similar pattern repeated in several instances during both June and July 2019. The European June and July heatwaves were the most lethal weather events in 2019, with more than 2500 excess deaths reported across France, Belgium and Netherlands^25^. Despite the very high temperatures, Spain reported a comparatively smaller number of heat-related deaths. While the lower death toll in Spain could be attributed to many factors, including the local population's higher resilience to heat, it is interesting that the heat indices peaked over the areas that reported the highest death toll. Further research is needed to understand the causes of higher excess mortality across northwest Europe.

### June 2021 Western United States heatwave

In June 2021, a ‘heat dome’ over the western United States and Canada resulted in record-breaking temperatures over much of British Columbia, Washington and Oregon^26,27^. We show the conditions from June 30, when maximum daily temperatures recorded over southeastern Washington state and northern Oregon were above 45 °C (113 °F) (Fig. 1d). Humidex and WBGT maps (Fig. 1g, h) confirm that parts of Washington and Oregon reached danger thresholds. However, these indices also show that the region experiencing dangerous conditions actually extends across British Columbia and Alberta and reaches into Northwest Territories. Humidex and WBGT maps suggest that: a) the most dangerous conditions were not concentrated solely where the highest temperatures were recorded and b) although experiencing very similar temperatures, parts of the Northwest Territories experienced different levels of heat danger. Interestingly, many provinces in Canada operate advanced alert systems that utilize both humidex and temperature^28^. During June 2021, conditions across most of Canada substantially exceeded predefined meteorological thresholds and heat alerts were raised. Despite this, the emergency response was deemed insufficient and over 600 heat-related deaths were recorded in British Columbia alone^29^. Issues reported in the aftermath of the event included an absence of clear protocols (resulting in delayed action by relevant public services), insufficient number of medical emergency crews available, infrastructure that is not adapted to hot weather, and a lack of understanding by the general population of the health hazards posed.

### June 2022 midwest United States heatwave

In the second week of June 2022, a heatwave affected the midwestern and southeast states of the United States and became infamous for causing the deaths of thousands of cattle across Kansas' plains^30^. On June 13, the highest daily temperatures (between 40 and 45 °C) were present over Kansas, Nebraska and Colorado, Texas, California and Arizona (Fig. 1g). According to humidex and WBGT values, very dangerous conditions were confirmed in Kansas, Nebraska and Texas, but also present in Arkansas, Missouri, Illinois, Indiana and Kentucky (Fig. 1h, i). The US National Weather Service (that considers heat index values in their heat warnings) had issued an excessive heatwave warning or heat advisories for over 60 million people across the southern parts of the country. Although the regions affected have had a long history of experiencing adverse heat effects, the June 2022 heatwave was a stark reminder that heatwaves affect animals too and also that for many ruminant species used in agriculture, the critical heat stress levels are lower than for humans^31^. The US Department of Agriculture has been working on establishing the Cattle Heat Stress Alert^32^, but it remains unclear to what extent farmers are utilizing the alert systems and if there are systems in place to protect the livestock from heat when needed. This event stresses the urgent need to consider heatwave impacts in their totality, including their effects on agriculture and ecosystems.

### May 2022 Pakistan-India heatwave

**Fig. 2:**
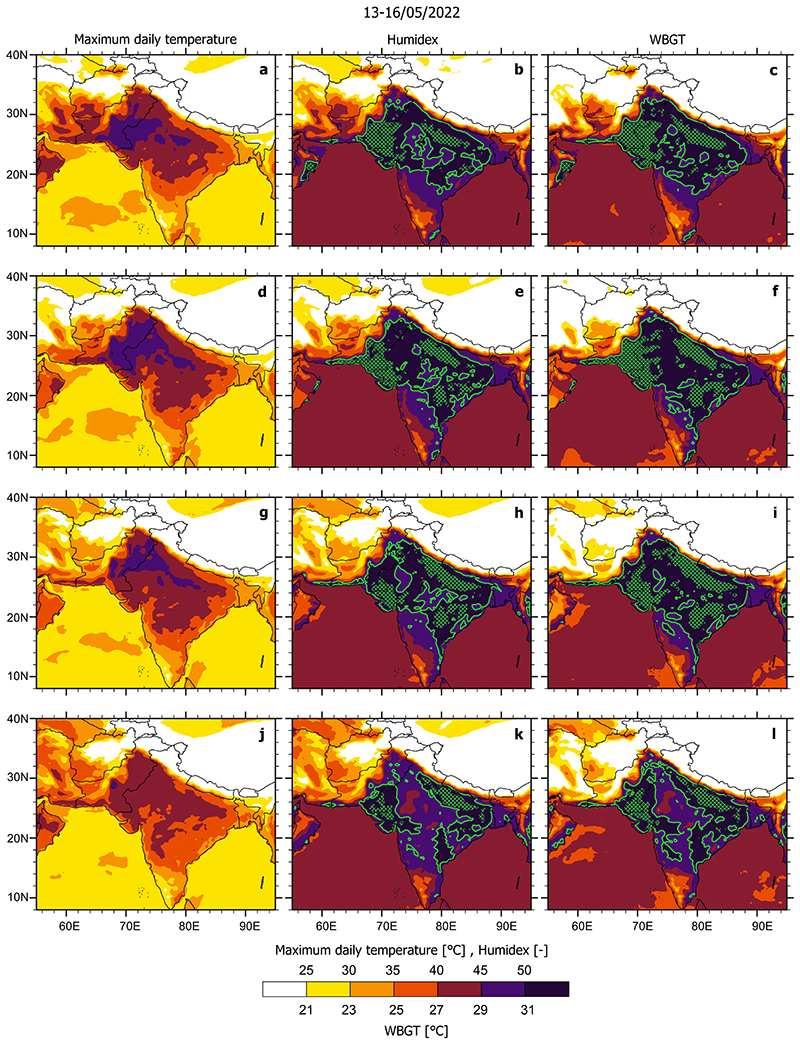
Maximum daily temperature [°C], humidex [unitless] and indoor Wet Bulb Globe Temperature (WBGT) [°C] during four selected days of the May 2022 Pakistan-India heatwave. Conditions shown are for the 13^th^ (**a-c**), 14^th^ (**d-f**), 15^th^ (**g-i**) and 16^th^ (**j-l**) of May 2022. Green contours indicate regions with humidex above 50 and indoor WBGT above 31 °C, where the critical threshold values defining dangerous heat stress conditions were exceeded; green stippled regions indicate humidex values above 54 and WBGT above 33 °C (these values have not been reached in the three heatwaves considered in Fig. 1).

In May 2022, a major heatwave hit Pakistan and northwest India, breaking numerous temperature records, with detrimental consequences for human health and agriculture^33^. Temperatures reached over 50 °C in the city of Jacobabad (Pakistan). Between May 13 and 16, temperatures above 45 °C were present in southern and western Pakistan and India's northwest regions. However, humidex and WBGT values suggest that very dangerous conditions occurred across India even over regions where maximum daily temperatures did not exceed 40 °C (Fig. 2). Despite experiencing temperatures 10 °C lower than those in the northwest India, high humidity from the Bay of Bengal resulted in what are considered very dangerous values for humidex and WBGT (above 54 and 33 °C, respectively) across much of the India's southeast. The May 2022 heatwave reached the thresholds thought to be at the very limits of human survival to extreme heat. Unfortunately, the lack of reliable medical data has thus far limited the lessons we could learn from this disaster.

Using the examples of recent major heatwaves, we illustrate how maps of maximum daily temperatures alone may lead to inadequate citizen perception of dangerous heatwave conditions. Complementing information about expected temperatures with information on health-tailored heat indices, while also actively educating the general public on their meaning, can help increase awareness and reduce the negative health impacts. Previous suggestions for improving awareness have included developing a heatwave ranking and naming systems^34^, as is the case with major storms and hurricanes. There is currently no agreed international system or protocol for naming or coordinating heatwave events, and heatwave alerts are country specific^35^. Physiological thresholds defined by the heat indices are a convenient tool because they can partly help overcome the regional-scale differences in definitions of heatwaves, making them easier to communicate to a broader audience. However, this still does not help the issue of no ‘one size fits all’ heat alert level, since the human body's response to heat varies depending on age, overall fitness and the existence of chronic conditions and that heat resilience can vary between different climate zones. Accordingly, sensitivity ranges for different target groups and potentially different thresholds for different climate zones would need to be carefully discussed and established. Finally, heatwave duration and its cumulative impacts are of extreme importance^36^ and ideally the total duration of extreme heat stress exposure should be considered. Given the large number of heat stress indices available and used operationally, selecting a universal one will not be trivial. This decision will require better communication between health and climate experts. They will need to identify the indices with the most robust medical data regarding physical impacts on the human body, that are also practical to apply operationally and easily communicated to the public.

Heatwaves have been responsible for 93% of climate-related deaths in Europe over the period 1970–2019^37^ and over 20 000 deaths in 2022^38^. Recent extreme heatwave episodes offer a glimpse into what is expected in the decades to come. In order to save lives, we must urgently take steps to adapt and educate the general population about the risks. Multi-disciplinary scientific engagement is needed to: i) decide on the most optimal heat stress descriptor to use in communication of dangerous heatwave conditions (in addition to temperatures) and devise strategies on how to educate the population about its meaning; ii) define the key information to convey to the public during heatwaves so they are adequately warned about when conditions are most dangerous and the mitigating measures that should be followed; iii) update the existing recommendations for local authorities during heatwaves, including criteria for when to close schools or stop sports activities, open cooling centers for socially vulnerable populations and ensure sufficient emergency response; iv) help raise the level of citizen engagement and create a “no one left behind” culture; and v) discuss alerts and warnings for safe keeping of animals and/or pets.

## Methods

### WBGT and Hu calculation

Indoor Wet Bulb Globe Temperature (WBGT) is calculated as weighted mean of wet bulb temperature WBT [°C] and air temperature Ta [°C] (assuming radiation-free indoor conditions where globe temperature equals the dry bulb temperature, as in Vecellio et al.^39^):

(1) WBGT=0.7*WBT+0.3*Ta

Wet bulb temperature WBT [°C] is calculated using the equation in Stull (2011)^40^:

(2) $$WBT=Ta*a\tan(0.151977*{\left(RH+8.313659\right)}^{0.5})+a\tan(Ta+RH)-a\tan(RH-1.676331)+0.00391838*a\tan(0.023101*RH)*{\left(RH\right)}^{1.5}-4.686035$$

where RH is the relative humidity in %, and Ta is the 2-m air temperature in [°C].

Relative humidity [%] was calculated from the 2-m dew point and air temperatures, Td and Ta, both expressed in °C:

(3) RH=100*exp(17.625*(Td/(243.04+Td)−Ta/(243.04+Ta)))

**Humidex (Hu) heat index** is calculated from 2-m air temperature Ta [°C] and 2-m dewpoint temperature Td [K] using the following expression (adapted from Barnett 2010^41^):

(4) Hu=Ta+3.394444*exp(19.833625−5417.7530/Td)−5.555556

### Critical thresholds for dangerous heat conditions

For Indoor Wet Bulb Globe Temperature (WBGT) we define critical thresholds for adverse health outcomes based on Fig. 2 in Vecellio et al.^39^, as: WBGT > 30–31 °C (87–88 °F) (defined for light ambulatory activity). These values are lower than previously used WBGT thresholds^20^.

For Humidex (Hu), we define strong heat stress conditions when Hu is between 45–54 °C and extreme heat stress conditions for Hu >54 °C (as in Schwingshackl et al.^20^).

### Reanalysis data

To calculate the Indoor Wet Bulb Globe Temperature (WBGT) and Humidex (Hu) heat indices, we utilized the maximum daily 2-m air and dew point temperatures (Ta, Td) from ERA5 reanalysis data^42^ (0.25° × 0.25° horizontal resolution). Since it is not possible to obtain Td values at the exact time when daily Ta maxima occurs, in our calculations we used both Ta and Td maximum daily values, meaning that the heat indices in Figs. 1, 2 represent an upper estimate of the respective maximum heat index value for that day. For comparison, in the Supplementary Discussion we also consider heat index values calculated using the maximum daily Ta and mean daily Td. The conclusion about different geographical distributions of temperature and heat index maxima remains unchanged (see Supplementary Fig. 1 and Supplementary Fig. 2).

## Supplementary Material

Supplementary Material

## Data Availability

The ERA5 meteorological data used in our analysis was retrieved from the Copernicus Climate Data Store: https://cds.climate.copernicus.eu/cdsapp#!/software/app-c3s-daily-era5-statistics?tab=overview. The source codes for the analysis of this study are available from the corresponding author upon request.
