# Supplementary material for: Importance of humidity for characterization and communication of dangerous heatwave conditions
Source: NPJ Clim Atmos Sci. Author manuscript; Available in PMC 2023 May 26. (PMC7614577; doi:10.1038/s41612-023-00346-x)
Supplement: Supplementary Material [file EMS176079-supplement-Supplementary_Material.pdf]

Supplementary Information for: "Importance of humidity for characterization and communication of dangerous heatwave conditions" by I. Cvijanovic, M. N. Mistry, J. D. Begg, A. Gasparrini and X. Rodó

Supplementary Discussion

In order to compare the impacts of using maximum vs. mean Td values in heat index calculations, in Supplementary Figure 1 and Supplementary Figure 2 we plot heat indices calculated using the maximum daily Ta and mean daily Td.

Since the daily mean Td is lower than the maximum daily Td, the resulting heat index values are lower when calculated using the mean daily Td (compare Supplementary Figure 1 and Supplementary Figure 2 with Figures 1 and 2). Importantly, the conclusion about different geographical distributions of temperature and heat index maxima remains unchanged.

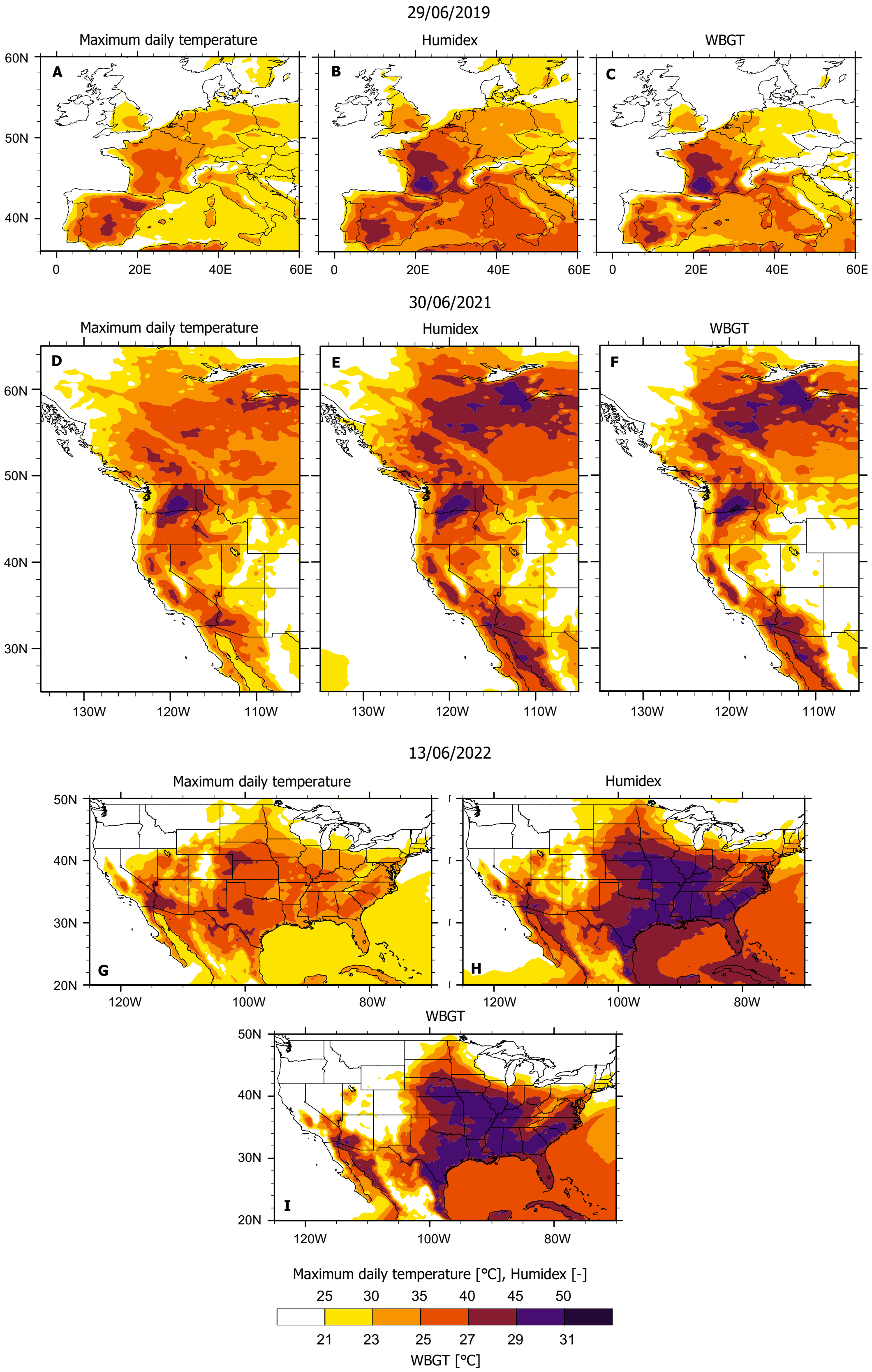

Supplementary Figure 1: Maximum daily temperature, humidex heat stress index and indoor Wet Bulb Globe Temperature (WBGT) during the selected days of three major heatwaves. (a, b, c): June 29th 2019 western Europe heatwave; (d, e, f): June 30th 2021 western United States heatwave; and (g, h, i): June 13th 2022 United States Midwest heatwave. Heat indices calculated using maximum daily temperature (Ta) and mean daily dew point temperature (Td).

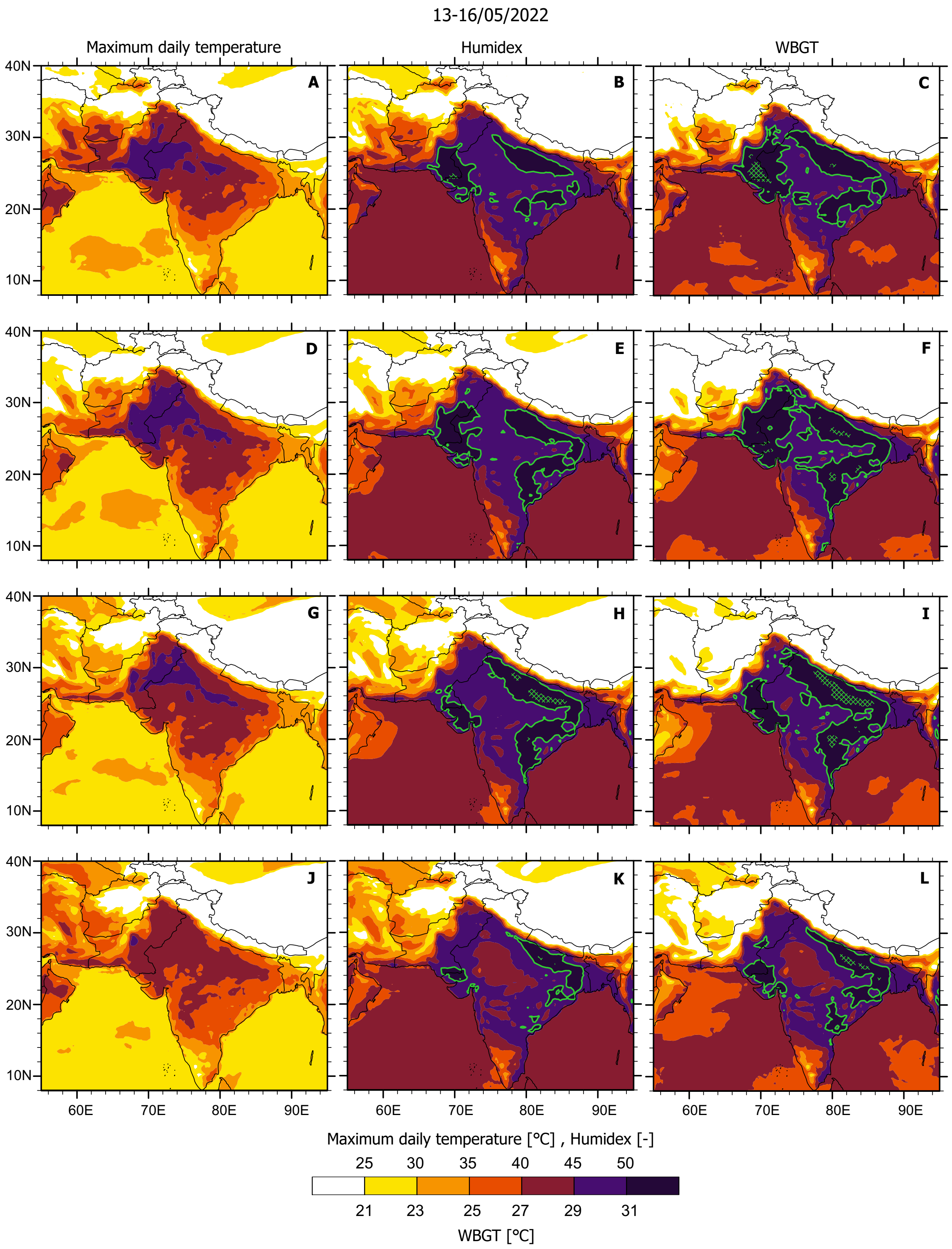

Supplementary Figure 2: Maximum daily temperature, humidex heat stress index and indoor Wet Bulb Globe Temperature (WBGT) during four selected days of the May 2022 Pakistan-India heatwave. Conditions shown are for the 13th (a, b, c), 14th (d, e, f), 15th (g, h, i) and 16th (j, k, l) of May 2022. Heat indices are calculated using maximum daily temperature (Ta) and mean daily dew point temperature (Td). Green contours indicate regions with humidex above 50 and indoor WBGT

above 31 °C, where the critical threshold values defining dangerous heat stress conditions were exceeded, stippled regions indicate humidex values above 54 and WBGT above 33 °C.
